# Impact of nighttime Rapid Response Team activation on outcomes of hospitalized patients with acute deterioration

**DOI:** 10.1186/s13054-018-2005-1

**Published:** 2018-03-14

**Authors:** Shannon M. Fernando, Peter M. Reardon, Sean M. Bagshaw, Damon C. Scales, Kyle Murphy, Jennifer Shen, Peter Tanuseputro, Daren K. Heyland, Kwadwo Kyeremanteng

**Affiliations:** 1 Division of Critical Care, Department of Medicine, University of Ottawa, Ottawa, ON Canada; 2 Department of Emergency Medicine, University of Ottawa, Ottawa, ON Canada; 3 Department of Critical Care Medicine, Faculty of Medicine and Dentistry, University of Alberta, Edmonton, AB Canada; 4 Department of Critical Care Medicine, Sunnybrook Health Sciences Centre, Toronto, ON Canada; 5 Interdepartmental Division of Critical Care, University of Toronto, Toronto, ON Canada; 6 Clinical Epidemiology Program, Ottawa Hospital Research Institute, Ottawa, ON Canada; 7 Bruyère Research Institute, Ottawa, ON Canada; 8 Division of Palliative Care, Department of Medicine, University of Ottawa, Ottawa, ON Canada; 9 Department of Critical Care Medicine, Queen’s University, Kingston, ON Canada

**Keywords:** Rapid Response Team, Intensive care unit, Critical care, Resuscitation

## Abstract

**Background:**

Rapid Response Teams (RRTs) are groups of healthcare providers that are used by many hospitals to respond to acutely deteriorating patients admitted to the wards. We sought to identify outcomes of patients assessed by RRTs outside standard working hours.

**Methods:**

We used a prospectively collected registry from two hospitals within a single tertiary care-level hospital system between May 1, 2012, and May 31, 2016. Patient information, outcomes, and RRT activation information were stored in the hospital data warehouse. Comparisons were made between RRT activation during daytime hours (0800–1659) and nighttime hours (1700–0759). The primary outcome was in-hospital mortality, analyzed using a multivariable logistic regression model.

**Results:**

A total of 6023 RRT activations on discrete patients were analyzed, 3367 (55.9%) of which occurred during nighttime hours. Nighttime RRT activation was associated with increased odds of mortality, as compared with daytime RRT activation (adjusted OR 1.34, 95% CI 1.26–1.40, *P* = 0.02). The time periods associated with the highest odds of mortality were 0600–0700 (adjusted OR 1.30, 95% CI 1.09–1.61) and 2300–2400 (adjusted OR 1.34, 95% CI 1.01–1.56). Daytime RRT activation was associated with increased odds of intensive care unit admission (adjusted OR 1.40, 95% CI 1.31–1.50, *P* = 0.02). Time from onset of concerning symptoms to RRT activation was shorter among patients assessed during daytime hours (*P* < 0.001).

**Conclusions:**

Acutely deteriorating ward patients assessed by an RRT at nighttime had a higher risk of in-hospital mortality. This work identifies important shortcomings in health service provision and quality of care outside daytime hours, highlighting an opportunity for quality improvement.

**Electronic supplementary material:**

The online version of this article (10.1186/s13054-018-2005-1) contains supplementary material, which is available to authorized users.

## Background

Patients admitted to the hospital wards are at risk of deterioration, and recognition of deterioration can be delayed [1, 2]. This puts these patients at increased risk of morbidity and mortality. Previous work has demonstrated that various adverse events, including cardiac arrest, unplanned intensive care unit (ICU) admission, and unexpected death, are usually preceded by objective signs of deterioration, often over the course of many hours [2]. In order to monitor such cases, many hospitals have instituted Rapid Response Teams (RRTs), which are a group of interprofessional critical care providers, often led by an intensivist, who immediately respond to ward patients experiencing clinical deterioration [3, 4]. RRTs do not routinely patrol wards, but are rather summoned by physicians or other health professionals who are caring for these patients, or in some instances, by patients’ families. Common causes for activation include respiratory distress, vital sign abnormalities (such as hypotension), or decreased level of consciousness; but healthcare providers are encouraged to activate RRT assistance if they are at all concerned about a patient’s condition [5].

The existing literature on the use of RRTs in clinical practice highlights many potential benefits. Most consistently, RRT use has been shown to reduce the incidence of in-hospital cardiac arrest, which has been attributed to early identification and treatment of at-risk patients [6–8]. Furthermore, a dose-response relationship between increasing RRT review rates and decreasing in-hospital cardiac arrest rates has been demonstrated [9]. Although the findings are less robust, RRTs have also been shown to improve overall hospital mortality, with particular benefit in reducing the incidence of unexpected in-hospital death [10]. RRTs are also thought to be useful for early involvement in end-of-life care and ensuring that patient treatment occurs within patient-specific limits of care [11].

At most centers, RRT availability exists at all times during the day. However, there may be differences in the amount and level of training of staff who are available at night [12]. Most hospital wards in Canada (including our centers) have reduced nursing-to-patient ratios at night, which has been shown to be associated with poorer performance in various quality-of-care indicators [13]. Additionally, attending physicians are less likely to be in the hospital during nighttime hours [14, 15]. There is often diurnal variability in RRT staffing. Whereas during the day the RRT may be staffed by a single dedicated attending physician, during the night RRTs may be more commonly composed of residents or emergency department (ED) physicians who are on shift at the time. There is also diurnal variation in patient-physician ratios, as well as in patient throughput (namely patient discharge) in the ICU, with a higher ratio and greater throughput found during the day [16], as is the case at our center. It is also well-accepted that staffing levels and expertise have an inverse relationship with patient outcomes [17]. We therefore attempted to determine whether nighttime RRT activation was associated with worse patient outcomes.

## Methods

Ethics approval for this study was obtained from The Ottawa Health Science Network Research Ethics Board (protocol number 20170016-01H).

### Study design, setting, and subjects

The study was performed at The Ottawa Hospital, a Canadian tertiary care hospital network that consists of two individual campuses, each with its own tertiary-level ICU. The Ottawa Hospital is a 1163-bed facility than handles over 160,000 emergency visits, 50,000 inpatients, and roughly 35,000 surgical cases annually. Each hospital has approximately 28–30 ICU beds. These are mixed medical and surgical ICUs. We analyzed prospectively collected data contained in the Ottawa Hospital Data Warehouse, which is a health administrative database widely used in previous research [18–20]. It contains information from several of the hospital’s information systems, including the patient registration system, clinical data repository, case-costing system, and patient discharge abstracts. Extensive assessments of data quality were performed during database development, and quality assurance initiatives to ensure completeness and accuracy of the data are conducted regularly [18].

We included all patients who met all of the following eligibility criteria: (a) over the age of 18 years; (b) admitted to either campus of The Ottawa Hospital; and (c) received RRT activation between May 1, 2012, and May 31, 2016. Patients with incomplete demographic or outcome data were excluded. We also excluded cases of routine scheduled RRT follow-up. We did not have data related to cases of cardiac arrest (because they involve a different response team). Patients were categorized by time of initial RRT activation. Patients with multiple activations during their admission were categorized on the basis of the time of their initial activation. Activations were classified as occurring during either daytime hours (0800–1659) or nighttime hours (1700–0759). At The Ottawa Hospital, RRTs during daytime hours are composed of an attending critical care specialist, a registered nurse, and a respiratory therapist. Nighttime RRT activations are managed by a resident physician on the critical care service, also with support from a registered nurse and respiratory therapist. An on-call intensivist is available but is not in-house. The same model is used at both sites. During daytime hours, most wards are under the direct supervision of an attending faculty physician. During nighttime hours, most hospital wards are under the supervision of resident physicians, with an attending physician available on-call. The RRT responds only to inpatients, outpatients experiencing distress (e.g., in radiology and endoscopy suites), or patients and family members requiring immediate care in hospital clinics or waiting rooms. The RRT does not respond to patients being assessed in the ED (who have not yet been admitted). Specific criteria for RRT activation at our center have been published previously [21]. They include concerns surrounding airway patency, tachypnea, systolic blood pressure ≤ 90 mmHg or ≥ 200 mmHg, decreased level of consciousness, and hypoxia, but healthcare providers are encouraged to activate the RRT for any reason of concern, even in the absence of objective changes in vital signs or laboratory values.

### Data collection

Patient information, including demographic data, comorbidities, previous ED visits, previous hospital admissions, and previous ICU admissions in the year prior to the index admission, was collected by registration clerical staff at the time of admission and stored in the data warehouse [18]. Information related to RRT activation is gathered by the RRT nursing staff at the time of patient assessment, and it is also stored in the data warehouse. This includes the most recent vital signs and laboratory values at the time of activation, reason for activation, admitting service, and whether the patient was admitted to the ICU. Data on latency time from onset of concerning symptoms or signs (e.g., hypotension, altered mental status) to RRT activation are also estimated by the RRT during assessment for quality improvement purposes. Outcome at hospital discharge (including death or disposition) and lengths of hospital and ICU stay are also stored in the data warehouse.

The primary outcome was in-hospital mortality, comparing patients assessed during daytime vs. nighttime hours. Secondary outcomes included ICU admission following RRT assessment and overall hospital length of stay.

### Statistical analysis

All statistical analyses were performed with commercially available statistical software packages (R version 3.3.3 [R Foundation for Statistical Computing, Vienna, Austria] and IBM SPSS Statistics version 24.0 [IBM, Armonk, NY, USA]). Data are presented as mean values (with SD) or as medians (with IQR), where indicated. Descriptive statistics were employed for between-group comparisons between daytime and nighttime hours, using Student’s *t* test for continuous variables and the χ^2^ test for categorical variables. In evaluating the outcomes of in-hospital mortality and ICU admission after RRT activation, we used multivariable logistic regression modeling to adjust for potential confounders, including patient characteristics (age, sex, comorbidities, comorbidity index, previous ED visits in the past year, previous hospital admissions in the past year, and previous ICU admissions in the past year), number of RRT activations, latency to RRT activation, most recent laboratory investigations at the time of RRT activation, and vital signs at the time of RRT activation. We also performed a secondary analysis using time of day as the exposure variable (with 1200–1300 as a reference, as reported previously [22]) to evaluate changes in adjusted mortality over the course of the day. We analyzed disposition of survivors at hospital discharge using a similarly constructed multivariable logistic regression model but restricted to patients originally admitted from home, assuming that patients initially admitted from peripheral acute or long-term care centers were likely to return to those centers. Adjusted ORs with 95% CIs and adjusted *P* values are provided. A *P* value ≤ 0.05 was taken to represent statistical significance.

## Results

### Patient cohort

The RRT was activated for 6132 discrete patients during the study period. Of these, 109 patients were excluded because of incomplete data, leaving 6023 patients in the study. A total of 2656 patients (44.1%) had calls that occurred during daytime hours (0800–1659), and 3367 (55.9%) had calls that occurred during nighttime hours (1700–0759).

Characteristics of the included patients, along with between-group comparisons between daytime and nighttime RRT activation are depicted in Table 1. The mean age of patients with RRT activation during daytime hours was 67.3 years (SD 16.7), whereas the mean age of those with RRT activation during nighttime hours was 67.9 years (SD 16.7) (*P* = 0.18). There was no significant difference in admission source (home, acute care facility transfer, long-term care facility transfer) between groups. There were no significant differences between groups in the median number of previous ED visits, hospital admissions, or ICU admissions in the past year.

**Table 1:** Characteristics of patients with daytime and nighttime Rapid Response Team activation

	Daytime hours (0800–1659) (*n* = 2656)	Nighttime hours (1700–0759) (*n* = 3367)	*P* value
Age, years, mean (SD)	67.3 (16.7)	67.9 (16.7)	0.18
Male sex, *n* (%)	1377 (53.0)	1816 (53.9)	0.91
Admission source, *n* (%)			0.97
Home	1854 (69.8)	2357 (70.0)	
Acute care facility transfer	296 (11.1)	379 (11.3)	
Long-term care facility transfer	240 (9.0)	306 (9.1)	
Unknown	266 (10.0)	325 (9.6)	
Comorbidities, *n* (%)			
Congestive heart failure	419 (15.8)	553 (16.4)	0.54
Arrhythmia	587 (22.1)	780 (23.2)	0.37
Valvular disease	80 (3.0)	100 (3.0)	0.90
Peripheral vascular disease	174 (6.6)	235 (7.0)	0.54
Hypertension	1007 (37.9)	1081 (32.1)	**< 0.001**
Chronic obstructive pulmonary disease	421 (15.9)	533 (15.8)	0.93
Diabetes mellitus	1183 (44.5)	1499 (44.5)	0.89
Renal failure	262 (9.9)	352 (10.5)	0.48
Liver disease	172 (6.5)	189 (5.6)	0.15
Metastatic cancer	376 (14.2)	523 (15.5)	0.15
Elixhauser comorbidity score, mean (SD)	9.0 (8.3)	9.1 (8.5)	0.51
Emergency department visits in past year, median (IQR)	1 (0–2)	1 (0–2)	0.20
Hospital admissions in past year, median (IQR)	0 (0–1)	0 (0–1)	0.35
ICU admissions in past year, median (IQR)	0 (0–0)	0 (0–0)	0.39

*ICU* Intensive care unit

Boldface font indicates statistical significance

### Characteristics of RRT activations

Between-group differences in characteristics of RRT activation calls are depicted in Table 2. There was a trend toward lower RRT use (number of RRT activations per 1000 hospital admissions, also termed the RRT “dose” [23]) during nighttime hours, though this difference was not statistically significant. Vital signs and laboratory values at the time of RRT activation are displayed. Reasons for RRT activation were significantly different between daytime and nighttime RRT calls (*P* < 0.001). Respiratory distress and tachycardia/arrhythmias were more common causes of activation at nighttime, whereas altered level of consciousness was more common during daytime hours. Calls arising from general nonspecific concerns about the patient were more common during the daytime. Nighttime RRT activation was associated with longer latency from symptom onset (*P <* 0.001). More RRT activations occurred within 1 h of symptom onset during daytime than during nighttime hours (81.0% vs. 73.2%, *P <* 0.001). There was no difference between groups in time to RRT response. A comparison of admitting services at the time of RRT activation is shown in Table 3. Some services (such as orthopedic surgery and hematology) more commonly requested RRT assistance during daytime hours, whereas others (such as neurosurgery and medical oncology) more often activated the RRT at nighttime.

**Table 2:** Characteristics of initial Rapid Response Team call for patients with daytime and nighttime Rapid Response Team activation

	Daytime hours (0800–1659) (*n* = 2656)	Nighttime hours (1700–0759) (*n* = 3367)	*P* value
Number of RRT activations during admission, median (IQR)	1 (1–1)	1 (1–1)	0.24
Patients with multiple RRT activations during admission, *n* (%)	473 (17.8)	629 (18.7)	0.38
RRT activations/1000 admissions	21.5	19.3	0.12
Most recent vital signs			
Systolic blood pressure, mmHg, mean (SD)	121 (29)	124 (32)	0.11
Diastolic blood pressure, mmHg, mean (SD)	70 (15)	71 (16)	**0.05**
Heart rate, beats/minute, mean (SD)	98 (30)	102 (30)	**< 0.001**
Temperature, °C, mean (SD)	36.8 (0.7)	36.8 (0.7)	0.28
Oxygen saturation, %, mean (SD)	94 (5)	94 (5)	0.66
Most recent laboratory values			
White blood cell count, ×10^9^/L, median (IQR)	10.1 (7.1–14.4)	10.7 (7.4–14.9)	**< 0.01**
Hemoglobin, g/L, mean (SD)	106.8 (22.9)	108.1 (22.6)	**0.03**
Platelets, ×10^9^/L, mean (SD)	225.4 (126.6)	229.6 (134.7)	0.22
Potassium, mmol/L, mean (SD)	4.1 (0.7)	4.1 (0.7)	0.87
Creatinine, μmol/L, median (IQR)	83 (60–142)	84 (60–142)	0.48
Urea, mmol/L, median (IQR)	7.4 (4.7–12.9)	7.6 (4.9–12.9)	0.18
Lactate, mmol/L, median (IQR)	2.2 (1.7–3.1)	2.2 (1.7–3.1)	0.92
Albumin, g/L, mean (SD)	27.1 (6.8)	26.9 (6.8)	0.20
INR, median (IQR)	1.2 (1.1–1.4)	1.2 (1.1–1.4)	0.62
Reason for call, *n* (%)			**< 0.001**
Respiratory distress	604 (22.7)	927 (27.5)	
Tachycardia/bradycardia/arrhythmia	395 (14.9)	643 (19.1)	
Altered level of consciousness	485 (18.3)	537 (15.9)	
Hypotension	356 (13.4)	388 (11.5)	
Hypertension	40 (1.5)	122 (3.6)	
Airway concern	90 (3.4)	124 (3.6)	
Seizure	30 (1.1)	30 (0.9)	
Worried about patient	336 (12.7)	304 (9.1)	
Other	152 (5.7)	152 (4.5)	
Unknown	168 (6.3)	140 (4.1)	
Latency from concerning symptom/sign onset to RRT activation			**< 0.001**
< 1 h	2151 (81.0)	2402 (73.2)	
1–4 h	437 (16.4)	667 (20.3)	
5–8 h	11 (0.4)	109 (3.3)	
9–12 h	8 (0.3)	26 (0.8)	
13–18 h	6 (0.2)	22 (0.7)	
19–24 h	4 (0.2)	18 (0.6)	
25–36 h	4 (0.2)	9 (0.3)	
37–48 h	2 (0.1)	2 (0.1)	
> 48 h	33 (0.4)	29 (0.9)	
RRT response time, minutes, median (IQR)	5 (3–7)	5 (3–7)	0.74

*Abbreviations: RRT* Rapid Response Team, *ICU* Intensive care unit, *INR* International normalized ratio

Boldface font indicates statistical significance

**Table 3:** Admitting service of patients with daytime and nighttime Rapid Response Team activation

	Daytime hours (0800–1659) (*n* = 2656)	Nighttime hours (1700–0759) (*n* = 3367)	*P* value
Service, *n* (%)			**< 0.001**
Surgical services			0.06
General surgery	285 (10.7)	382 (11.4)	
Orthopedic surgery	240 (9.0)	248 (7.4)	
Vascular surgery	116 (4.4)	136 (4.0)	
Neurosurgery	85 (3.2)	152 (4.5)	
Thoracic surgery	81 (3.1)	103 (3.1)	
Obstetrics and gynecology	66 (2.5)	73 (2.2)	
Urology	64 (2.4)	82 (2.4)	
Otolaryngology	27 (1.0)	42 (1.3)	
Plastic surgery	4 (0.2)	9 (0.3)	
Nonsurgical Services			**< 0.001**
General internal medicine	715 (26.9)	934 (27.7)	
Hematology	208 (7.8)	197 (5.9)	
Intensive care	144 (5.4)	140 (4.2)	
Medical oncology	112 (4.2)	230 (6.8)	
Family medicine	103 (3.9)	130 (3.9)	
Nephrology	92 (3.5)	112 (3.3)	
Radiation oncology	83 (3.1)	79 (2.4)	
Neurology	76 (2.9)	96 (2.9)	
Respirology	52 (2.0)	91 (2.7)	
Psychiatry	49 (1.8)	53 (1.6)	
Cardiology	31 (1.2)	48 (1.4)	
Rehabilitation	16 (0.6)	19 (0.6)	
Gastroenterology	7 (0.3)	11 (0.3)	

Boldface font indicates statistical significance

### Nighttime RRT activation and patient mortality

**Fig. 1:**
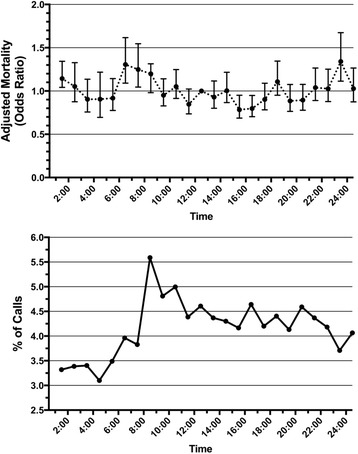
Percentage of total calls and adjusted in-hospital mortality by time of day of Rapid Response Team activation (using 1200 as a reference)

Logistic regression using multivariable models with adjustment for patient variables demonstrated that nighttime RRT activation was associated with significantly higher odds of in-hospital mortality (adjusted OR 1.34, 95% CI 1.26–1.40) (Table 4). The logistic regression model, including all covariables, is depicted in Additional file 1. Daytime RRT activation was associated with significantly higher odds of ICU admission (adjusted OR 1.40, 95% CI 1.30–1.48). No differences in overall hospital length of stay (*P* = 0.92) or disposition of survivors (*P* = 0.47) were found between groups. The frequency of RRT calls, with associated risk of mortality across the day, is depicted graphically in Fig. 1. Of note, the frequency of RRT activation increases dramatically at approximately 0800, shortly after the beginning of daytime hours. Two statistically significant time periods were associated with increased adjusted odds of in-hospital mortality: 0600–0700 (adjusted OR 1.30, 95% CI 1.09–1.61) and 2300–2400 (adjusted OR 1.34, 95% CI 1.01–1.56). A sensitivity analysis excluding patients admitted to the ICU was performed (Additional file 2). There were no differences in mortality or hospital length of stay between groups.

**Table 4:** Outcomes in patients with daytime and nighttime Rapid Response Team activation

	Daytime hours (0800–1659) (*n* = 2656)	Nighttime hours (1700–0759) (*n* = 3367)	Adjusted OR (95% CI)	Adjusted*P* value
In-hospital mortality, *n* (%)	772 (29.6)	1061 (32.0)	1.34^a^ (1.26–1.40)	**0.02** ^a^
Admitted to ICU, *n* (%)	760 (30.5)	948 (27.8)	1.40^a^ (1.30–1.48)	**0.02** ^b^
Hospital length of stay, days, median (IQR)	14 (7–29)	14 (7–28)		0.92
Survivors discharged to home, *n* (%)^b^	903 (48.7)	1131 (48.0)	1.01^a^ (0.91–1.07)	0.47

*ICU* Intensive care unit

^a^OR and *P* value were adjusted for age, sex, comorbidities, previous emergency department visits in the past year, previous hospital admissions in the past year, previous ICU admissions in the past year, total number of Rapid Response Team (RRT) calls, latency to RRT activation, laboratory values at the time of RRT activation, vital signs at the time of RRT activation, reason for RRT activation, and admitting service, using multivariate logistic regression

^b^Analysis includes only patients originally admitted from home

Boldface font indicates statistical significance

## Discussion

We found that RRT activation during nighttime hours (1700–0759) was associated with an increased risk of in-hospital mortality compared with daytime (0800–1659) activation. The two time periods associated with highest risk of in-hospital mortality were 0600–0700 and 2300–2400. These times correspond to workforce shift changes at our institution, particularly with regard to ward nursing care. Patients who were seen by the RRT during nighttime were less commonly admitted to the ICU. These findings were evident even when adjusted for many possible confounding patient factors. Nighttime RRT activations had increased latency from symptom onset compared with daytime RRT activations.

Our results demonstrate variability in outcomes with RRT activation over the course of the day. Our findings are consistent with a recent large database study in the United States [22] and a smaller study in Australia [24], which also found increased mortality with overnight RRT activation and an increased adjusted risk of mortality for activations between 0700 and 0800. These studies also found that the lowest frequency of calls occurred in the early morning (immediately preceding the time associated with the highest risk of mortality) and that the highest frequency of calls occurred between 0800 and 0900. They suggested that this may be due to workforce shift changes, and the investigators hypothesized that there may be increased delay to RRT activation overnight, but they did not have this data available to them. Our study adds to this work not only by supporting these findings but also by adjusting for more confounding variables. We found that nighttime RRT activation was in fact associated with prolonged delays to activation. There are likely many reasons for this, including reduced patient/nursing ratios, coupled with fewer and less-experienced physicians (on both the ward as well as the RRT), on hospital wards at night. Similar work has demonstrated that RRT activation more commonly occurs after nurse shift change and vital sign checks [25, 26], which may explain the dramatic increase in frequency after 0800.

Understanding why these differences in outcomes occur is paramount in identifying strategies for quality improvement in recognition of deteriorating patients on hospital wards, as well as for improvement in RRT function. Diurnal differences in patient outcomes have been reported in various disease processes, including cardiac arrest, trauma, and myocardial infarction [27–29]. As mentioned, this may be related to known differences in the amount and experience of staffing (both physicians and nurses) overnight, particularly in critical care settings [14, 16, 17, 30, 31]. Although we found no differences in patient characteristics or most recent vital signs or laboratory work between daytime and nighttime RRT activation, patients admitted to the ICU during early morning hours tend to be older and sicker than those admitted later in the day [32]. We found that RRT activation during the daytime was more likely to result in ICU admission than activations occurring during nighttime. Because delayed ICU admission is a factor associated with worse outcomes in critically ill patients [33], moving these patients to a critical care setting may have partially accounted for the improved survival seen in activations during daytime hours. This may have been affected by bed availability because fewer ICU beds are available at night [34].

Importantly, we found that nighttime RRT activation was associated with prolonged latency from the onset of concerning symptoms and signs. Delays in RRT activation have been shown to be associated with worse outcomes [35, 36]. These increased delays may occur because of impaired recognition and/or monitoring of clinical deterioration. Diurnal variation in shift times and durations also influence staff performance, which has been shown to decline during the night [37]. In tertiary care centers, most nighttime services are under the supervision of medical residents, who not only have less experience than their attending physician counterparts but also often work many consecutive hours, with concomitant sleep deprivation. These factors together result in poorer cognitive performance [38, 39] and may contribute to reduced recognition of clinical deterioration among admitted patients. Additionally, these residents may be busy performing other clinical duties, owing to a reduced number of personnel available during nighttime hours [16]. In keeping with this, we found a trend toward lower “RRT dose” during nighttime hours, though the difference was not statistically significant. Increasing RRT dose has been associated with a reduction in adverse events among hospitalized patients [23], and lower dosing has previously been demonstrated during nighttime hours [40].

This study has several strengths, including a large sample size as well as data related to various patient and RRT activation variables. The main outcomes were assessed using logistic regression models to control for the influence of important patient factors. However, there are several limitations that hinder the generalizability of our results. First, the observational nature of our dataset allows detection of associations but does not reveal the causal pathways that lead to increased mortality risk after nighttime RRT activations. Second, although the data were gathered from two different hospitals, the hospitals are located within the same city and use the same RRT model, which may limit the generalizability of the results. However, as mentioned, our findings are similar to those of recent studies in the United States [22] and Australia [24]. Although we attempted to control for many confounding patient and RRT factors, including laboratory values and vital signs, there are some factors that we were unable to include in the model, such as the degree of acute physiological derangement. We were also unable to compute the “Score to Door Time,” which is an indicator of quality of healthcare delivery, for patients requiring ICU admission [41]. Finally, although we were able to gather data related to the latency of RRT response, we did not have data related to time until treatment, which may have been delayed during nighttime RRT activations.

## Conclusions

We found that nighttime RRT activation was associated with increased mortality, decreased ICU admission, and increased latency to activation in hospitalized patients with acute deterioration. These findings underscore important shortcomings in both the recognition of these patients by ward staff and the function of RRTs, and they serve as an important avenue for future quality improvement.

## Additional files

Additional file 1: Multivariate logistic regression analysis of factors associated with in-hospital mortality (*n* = 6023). Multivariate logistic regression variables, with associated ORs and 95% CIs. (DOCX 136 kb)

Additional file 2: Time of rapid response team activation and patient outcomes among patients not admitted to the intensive care unit. Subgroup analysis of outcomes among patients not admitted to the intensive care unit. (DOCX 77 kb)

## Abbreviations

ED: Emergency department
ICU: Intensive care unit
INR: International normalized ratio
RRT: Rapid Response Team
