# Supplementary material for: Impact of nighttime Rapid Response Team activation on outcomes of hospitalized patients with acute deterioration
Source: Crit Care. 2018 Mar 14;22:67. doi: 10.1186/s13054-018-2005-1 (PMC5851273; doi:10.1186/s13054-018-2005-1)
Supplement: Supplementary file 1 — Multivariate logistic regression analysis of factors associated with in-hospital mortality (n = 6023). Multivariate logistic regression variables, with associated ORs and 95% CIs. (DOCX 136 kb) [file 13054_2018_2005_MOESM1_ESM.docx]

**Additional File 1**

| **Variable** | **Odds Ratio** | **95% CI** | ***P Value*** |
| --- | --- | --- | --- |
| Age | 1.03 | 0.72-1.41 | 0.25 |
| Male Sex | 1.03 | 0.86-1.13 | 0.16 |
| ED Visits in Past Year | 0.96 | 0.74-1.12 | 0.41 |
| Hospital Admissions in Past Year | 1.04 | 0.92-1.15 | 0.29 |
| ICU Admissions in Past Year | 0.88 | 0.76-1.06 | 0.21 |
| Admission Source | 1.02 | 0.74-1.29 | 0.20 |
| Past Medical History | | | |
| Congestive Heart Failure | 0.95 | 0.85-1.05 | 0.79 |
| Arrhythmia | 0.80 | 0.59-1.12 | 0.38 |
| Valvular Disease | 1.08 | 0.88-1.19 | 0.51 |
| Peripheral Vascular Disease | 1.03 | 0.93-1.17 | 0.54 |
| Hypertension | 0.86 | 0.80-1.05 | 0.62 |
| Chronic Obstructive Pulmonary Disease | 1.10 | 0.93-1.16 | 0.27 |
| Diabetes Mellitus | 0.96 | 0.88-1.07 | 0.43 |
| Renal Failure | 0.91 | 0.82-1.09 | 0.35 |
| Liver Disease | 1.06 | 0.89-1.17 | 0.41 |
| Metastatic Cancer | 1.08 | 0.86-1.22 | 0.23 |
| Elixhauser Comorbidity Index | 1.03 | 0.84-1.10 | 0.43 |
| RRT Activation During Night-time Hours | 1.34 | 1.26-1.40 | **0.02** |
| Total Number of RRT Calls | 1.04 | 0.83-1.15 | 0.33 |
| > 1 Hour of Latency to RRT Activation | 1.27 | 1.15-1.34 | **0.04** |
| Most Recent Vital Signs | | | |
| Systolic Blood Pressure, mmHg | 1.05 | 0.89-1.17 | 0.22 |
| Diastolic Blood Pressure, mmHg | 1.01 | 0.91-1.10 | 0.26 |
| Heart Rate, Beats/Min. | 1.09 | 0.97-1.16 | 0.18 |
| Temperature, Degrees Celsius | 0.94 | 0.79-1.18 | 0.28 |
| Oxygen Saturation, % | 0.89 | 0.76-1.07 | 0.36 |
| Most Recent Blood Work | | | |
| White Blood Cell Count, x10^9^/L | 1.07 | 0.97-1.13 | 0.55 |
| Hemoglobin, g/L | 0.89 | 0.80-0.94 | 0.38 |
| Platelets, x10^9^/L | 0.93 | 0.79-1.05 | 0.31 |
| Potassium, mmol/L | 1.06 | 0.97-1.19 | 0.45 |
| Creatinine, µmol/L | 1.18 | 0.98-1.41 | 0.12 |
| Urea, mmol/L | 1.15 | 0.96-1.34 | 0.14 |
| Lactate, mmol/L | 1.30 | 0.98-1.55 | 0.10 |
| Albumin, g/L | 0.92 | 0.82-1.15 | 0.47 |
| INR | 1.07 | 0.93-1.18 | 0.48 |
| Reason for Call | 1.04 | 0.85-1.20 | 0.28 |
| Admission Service | 1.06 | 0.87-1.27 | 0.24 |

***Additional File 1:*** Multivariate logistic regression analysis of factors associated with in-hospital mortality (n = 6,023). Area under receiver operating characteristics curve = 0.84. Hosmer-Lemeshow *P* value = 0.29.

Abbreviations: ED = Emergency Department; ICU = Intensive Care Unit; RRT = Rapid Response Team; IQR = Interquartile range; CI = Confidence Interval; INR = International normalized ratio
