# Supplementary material for: Impact of nighttime Rapid Response Team activation on outcomes of hospitalized patients with acute deterioration
Source: Crit Care. 2018 Mar 14;22:67. doi: 10.1186/s13054-018-2005-1 (PMC5851273; doi:10.1186/s13054-018-2005-1)
Supplement: Supplementary file 2 — Time of rapid response team activation and patient outcomes among patients not admitted to the intensive care unit. Subgroup analysis of outcomes among patients not admitted to the intensive care unit. (DOCX 77 kb) [file 13054_2018_2005_MOESM2_ESM.docx]

**ADDITIONAL FILE 2:** **Time of Rapid Response Team Activation and Patient Outcomes Among Patients Not Admitted to the Intensive Care Unit**

|  | **Daytime Hours 0800-1659**  **(n = 1896)** | **Night-time Hours 1700-0759**  **(n = 2419)** | **Adjusted Odds Ratio (95% CI)** | **Adjusted**  ***P*-Value** |
| --- | --- | --- | --- | --- |
| In-Hospital Mortality, n (%) | 507 (26.7) | 675 (27.9) | 0.96^a^  (0.92-1.06) | 0.41^a^ |
| Hospital Length of Stay, days, median (IQR) | 13 (6-26) | 13 (6-25) |  | 0.61 |
| Survivors Discharged Home, n (%)^b^ | 681 (49.0) | 839 (48.1) | 1.01^a^  (0.92-1.06) | 0.57 |

***Additional Table 2:*** Time of Rapid Response Team Activation and Patient Outcomes Among Patients Not Admitted to the Intensive Care Unit

^a^Odds ratio and *P*-value were adjusted for age, sex, comorbidities, previous Emergency Department visits in the past year, previous hospital admissions in the past year, previous ICU admissions in the past year, total number of RRT calls, latency to RRT activation, laboratory values at the time of RRT activation, vital signs at the time of RRT activation, reason for RRT activation, and admitting service, using multivariate logistic regression.

^b^Analysis only includes patients originally from home.

Abbreviations: RRT = Rapid Response Team; ICU = Intensive Care Unit; IQR = Interquartile range; CI = Confidence Interval
